# High-performance solid-state electrochemical thermal switches with earth-abundant cerium oxide

**DOI:** 10.1126/sciadv.ads6137

**Published:** 2025-01-01

**Authors:** Ahrong Jeong, Mitsuki Yoshimura, Hyeonjun Kong, Zhiping Bian, Jason Tam, Bin Feng, Yuichi Ikuhara, Takashi Endo, Yasutaka Matsuo, Hiromichi Ohta

**Affiliations:** ^1^ Research Institute for Electronic Science, Hokkaido University, N20W10, Kita, Sapporo 001-0020, Japan.; ^2^ Graduate School of Information Science and Technology, Hokkaido University, N14W9, Kita, Sapporo 060-0814, Japan.; ^3^ Institute of Engineering Innovation, The University of Tokyo, 2-11-16 Yayoi, Bunkyo, Tokyo 113-8656, Japan.

## Abstract

Thermal switches, which electrically turn heat flow on and off, have attracted attention as thermal management devices. Electrochemical reduction/oxidation switches the thermal conductivity (κ) of active metal oxide films. The performance of the previously proposed electrochemical thermal switches is low; the on/off κ-ratio is mostly less than 5, and the κ-switching width is less than 5 watts per meter kelvin. We used a CeO_2_ thin film as the active layer deposited on a solid electrolyte YSZ substrate. When the CeO_2_ thin film was reduced once (off-state) and then oxidized (on-state), κ was about 2.2 watts per meter kelvin in the most reduced state, and κ increased with oxidation to 12.5 watts per meter kelvin (on-state). This reduction (off-state)/oxidation (on-state) cycle was repeated 100 times, and the on/off κ-ratio was 5.8, and the κ-switching width was 10.3 watts per meter kelvin. The CeO_2_-based solid-state electrochemical thermal switches would be potential devices for thermal shutters and thermal displays.

## Teaser

High-performance thermal switches that control heat flow electrochemically using earth-abundant materials have been realized.

## INTRODUCTION

Because of low energy conversion efficiency, two-thirds of primary energy, such as oil and coal, is lost as waste heat. In particular, low- to medium-temperature (100° to 300°C) waste heat is the most difficult to reuse (*1*). Thermoelectric energy conversion technology, which directly converts a temperature difference into electricity, is one solution, but its efficiency is low in this temperature range in air (*2*, *3*). On the other hand, thermal management technologies such as thermal diodes and thermal transistors (hereinafter referred to as thermal switches) have recently attracted attention (*4*). Thermal diodes rectify heat flow, and thermal switches turn heat flow on and off. We expect that thermal displays that visualize heat contrast using infrared cameras can be realized using thermal switches. Similar to liquid crystal displays, the transmission of backlight (infrared energy from waste heat) can be controlled by thermal switches. Therefore, thermal switches may be useful for reusing waste heat as an infrared source.

Several types of thermal switches have been proposed. First, theorists proposed thermal switches that use heat-induced modulation of thermal conductivity (κ) modulation (*5*–*7*). In 2014, Ben-Abdallah and Biehs (*6*) proposed electrical switching of the κ of VO_2_, which exhibits an insulator-to-metal transition at 68°C in bulk form. Unfortunately, the idea was not realized because the κ of VO_2_ does not change when the temperature reaches the transition temperature (*8*, *9*). Since then, several thermal switches have been proposed that exploit the κ modulation by the electrochemical reaction of active materials: LiCoO_2_/Li_1−*x*_CoO_2_ (*10*), MoS_2_/Li*_x_*MoS_2_ (*11*), SrCoO_3_/SrCoO_2.5_/HSrCoO_2.5_ (*12*), MoS_2_/MoS_2_-organic molecular hybrid (*13*), SrCoO_3_/SrCoO_2_ (*14*–*16*), La_0.5_Sr_0.5_CoO_3_/La_0.5_Sr_0.5_CoO_2.5_ (*17*), and LaNiO_3_/LaNiO_2.72_ (*18*). In addition, Tomko *et al*. (*19*) reported κ switching in topologically networked bioinspired materials. Castelli *et al*. (*20*) reported three-terminal magnetic thermal switches that control heat flow by changing the touch/removal of the metal bridge using a magnet. Liu *et al*. (*21*) demonstrated low-voltage controlled thermal switching in antiferroelectric PbZrO_3_ thin films. Li *et al*. (*22*) developed a three-terminal thermal switch that electrostatically controls the κ of an organic molecule-based self-assembled monolayer. In addition, Hartquist *et al*. (*23*) demonstrated that the deformation of polymer materials can be used to tune κ. Among these various proposed thermal switches, in this study we focused on oxide-based solid-state electrochemical thermal switches (*14*).

Oxide-based solid-state electrochemical thermal switches have several disadvantages, including low operating speed, high operating temperature, low performance, and the presence of rare metal elements. First, compared to electrostatic modulation (*22*), electrochemical modulation takes time to turn thermal switches on and off. Although it is necessary to modulate the κ of thick active materials for practical applications such as thermal displays, electrostatic modulation can only modulate the interface between the gate and the active materials. With electrochemical thermal switches, the κ of the entire film can be modulated. This is one advantage of electrochemical thermal switches. Second, the operating temperature of solid-state electrochemical thermal switches is high (~280°C) compared to liquid electrolyte–based electrochemical thermal switches, which can be operated at room temperature. However, we want to use the low-to-medium temperature (100° to 300°C) waste heat exhausted into the air as the backlight of thermal displays, so a relatively high operating temperature is not a problem. In addition, the thermal switches must be stable over the temperature range and must not contain any liquid. Since most oxide materials are generally stable in the temperature range in air, we chose oxides as the active materials. Third, the performance such as the on/off κ-ratio and κ-switching width of oxide-based solid-state electrochemical thermal switches is lower compared to the other types of thermal switches. According to our previous studies, materials with high electrical conductivity are promising as active materials to improve the κ-switching width of solid-state thermal switches, because such materials have high electron κ (*15*). Recently, we have focused on LaNiO_3_ as an active material of solid-state thermal switches because LaNiO_3_ exhibits very high electrical conductivity (~10,000 S cm^−1^) and high κ [11 W m^−1^ K^−1^ (*24*)] at room temperature. We have achieved LaNiO_3_-based thermal switches with a large κ*-*switching width (~4.3 W m^−1^ K^−1^) by exploiting its high electron κ of ~3.1 W m^−1^ K^−1^ (*18*). Although this widens the κ-switching width, the width is still less than 5 W m^−1^ K^−1^. Therefore, we are still searching for oxide-based solid-state electrochemical thermal switches with a large on/off κ-ratio and a much wider κ-switching width. Fourth, the previously reported oxide-based thermal switches contain rare metal elements of Co and Ni. The use of rare metal elements is not suitable for sustainable development.

In this study, we focused on the electrochemical reduction/oxidation of CeO_2_ as an active material for thermal switches. CeO_2_ is an abundant material that is widely used in practical applications such as polishing powders, catalysts, and sunscreens. The crystal structure of CeO_2_ is a simple cubic fluorite (space group: *Fm*$\bar{3}$*m*), with a lattice parameter of 0.541 nm. CeO_2_ films can be grown heteroepitaxially on YSZ single crystals (lattice parameter, *a* = 0.515 nm) with a lattice mismatch of about +5%. Bulk CeO_2_ exhibits a high κ value (~14 W m^−1^ K^−1^) at room temperature (*25*–*27*), which is higher than that of SrCoO_3_ [3.8 W m^−1^ K^−1^ (*14*)] and LaNiO_3_ [11 W m^−1^ K^−1^ (*24*)]. Moreover, CeO_2_ can be reduced thermochemically (*28*–*32*) and electrochemically (*33*). Oxygen-deficient homologous phases of Ce*_n_*O_2*n*−2_ (*n* ≥ 4, integer) are known (*28*–*32*). In 2014, Khafizov *et al*. (*34*) obtained theoretical results indicating that CeO_1.735_ (*n* ~ 7 in Ce*_n_*O_2*n*−2_) would have a very low κ value (~1.2 W m^−1^ K^−1^), which is close to the amorphous limit for thermal transport (κ ~ 0.9 W m^−1^ K^−1^) (*35*). Therefore, we expected that CeO_2_-based thermal switches would have a large on/off κ-ratio of >10 and a large κ-switching width ~13 W m^−1^ K^−1^, which would be outstanding among the reported oxide-based thermal switches.

Here, we show that CeO_2_-based solid-state electrochemical thermal switches exhibit a relatively large on/off κ-ratio of 5.8 and a wide κ-switching width of ~10.3 W m^−1^ K^−1^ (on-state: 12.5 W m^−1^ K^−1^, off-state: 2.2 W m^−1^ K^−1^). This performance is outstanding among reported oxide-based solid-state electrochemical thermal switches. Since CeO_2_ is an earth-abundant material, the present CeO_2_-based solid-state electrochemical thermal switches would have a potential for use in practical thermal management devices such as thermal displays.

## RESULTS

### Electrochemical reduction/oxidation of cerium oxide films

**Fig. 1.**
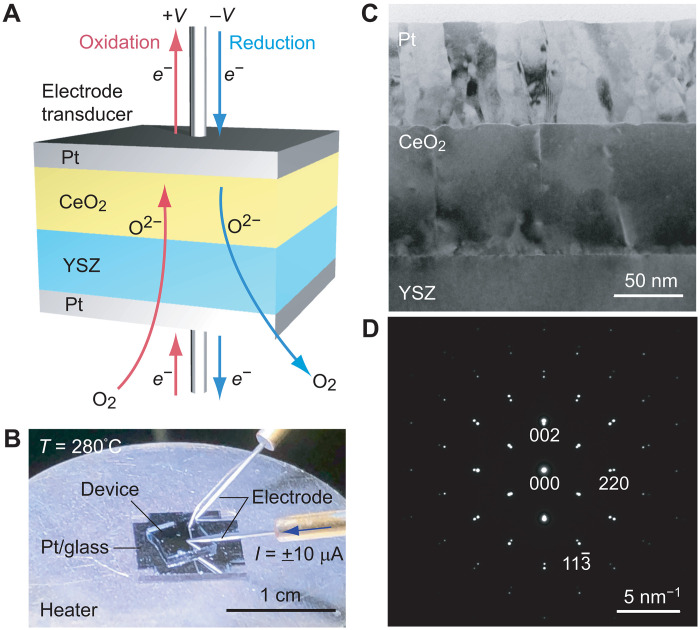
A CeO_2_-based solid-state thermal switch. (**A**) Schematic device structure of a CeO_2_-based solid-state electrochemical thermal switch, which consists of a 103-nm-thick CeO_2_ epitaxial film grown on a 0.5-mm-thick YSZ substrate, sandwiched between two Pt films. When a negative/positive voltage is applied to the top Pt electrode, electrochemical reduction/oxidation of the CeO_2_ layer occurs. The top Pt electrode is also used as a transducer for the thermal conductivity measurements. (**B**) Photograph of a CeO_2_-based thermal switch operating at 280°C in the air. The surface area of the device is ~25 mm^2^. A constant current of +10 μA (−10 μA) was applied for oxidation (reduction). (**C**) Cross-sectional TEM image of the thermal switch. The Pt/CeO_2_/YSZ trilayer structure is clearly visible. (**D**) Selected area electron diffraction pattern. Diffraction spots of the CeO_2_ film are seen together with those of the YSZ. The crystallographic orientation is (001)[110]_CeO2_ || (001)[110]_YSZ_.

First, we fabricated CeO_2_-based thermal switches (Fig. 1). Details of the device fabrication procedure are described in text S1 and Materials and Methods. Figure 1A shows the device structure of a CeO_2_-based solid-state electrochemical thermal switch, which consists of a 103-nm-thick CeO_2_ epitaxial film on a 0.5-mm-thick YSZ substrate sandwiched between Pt films. The electrochemical reduction/oxidation treatments were performed at 280°C in the air (Fig. 1B). A 5 mm–by–5 mm device was placed on a heater (280°C), and a negative/positive voltage was applied to the top Pt electrode. The trilayer structure was visible in the cross-sectional transmission electron microscopy (TEM) image (Fig. 1C). The selected area electron diffraction pattern (Fig. 1D) indicates that the CeO_2_ film was heteroepitaxially grown on YSZ with a cube-on-cube relationship.

**Fig. 2.**
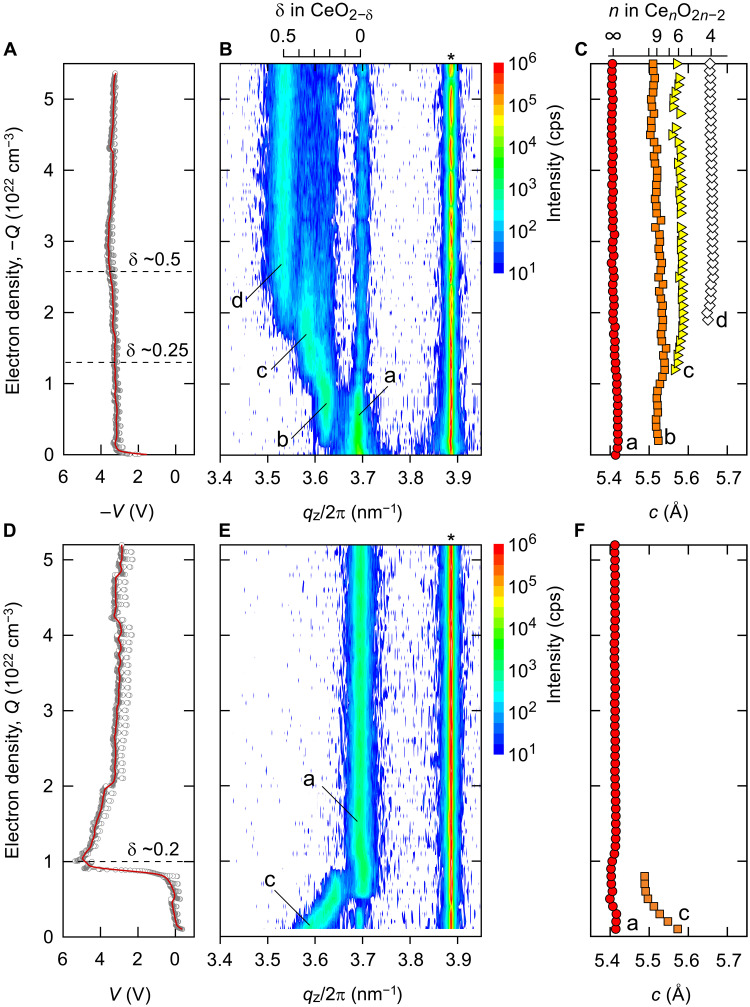
Electrochemical reduction/oxidation treatment of a CeO_2_-based solid-state electrochemical thermal switch. (**A** and **D**) Changes in the applied voltage during reduction and oxidation treatments. During electrochemical reduction, the absolute value of the applied voltage is almost constant (~3.3 V). In contrast, the applied voltage increases markedly from 0 to 5 V during oxidation. (**B** and **E**) Changes in the out-of-plane XRD patterns. After reduction treatment, the diffraction peak of 002 CeO_2−δ_ (labeled b, c, and d) appears sequentially in addition to the diffraction peak of 002 CeO_2_ (labeled a) with 002 YSZ (*). After oxidation treatment, the diffraction peak of 002 CeO_2−δ_ (c) shifts to the larger *q*_z_/2π side and lastly disappears. (**C** and **F**) Lattice parameters, *c*, of the CeO_2_ (a) and CeO_2−δ_ (b, c, and d) extracted from the out-of-plane XRD patterns. The *c* of the CeO_2_ (a) is almost constant (0.541 nm). From the *c*, a, b, c, and d phases are found to be CeO_2_, Ce_9_O_16_, Ce_6_O_10_, and Ce_2_O_3_, respectively. cps, counts per unit.

The electrochemical reduction/oxidation treatments were performed by applying a constant current of −10 μA/+10 μA (Fig. 2, A and D). These treatments were performed by applying an electron density *Q* of 1 × 10^21^ cm^−3^ each time. During electrochemical reduction (Fig. 2A), the absolute value of the applied voltage remains almost constant (~3.3 V). We repeated the reduction treatment until the total *Q* reached −5.5 × 10^22^ cm^−3^, corresponding to a total reduction time of 2255 s. In contrast, the voltage required for initial oxidation was negative, reflecting spontaneous oxidation (Fig. 2D). When *Q* exceeds ~7 × 10^21^ cm^−3^, the required voltage increases markedly from ~0 to ~5 V. The required voltage became saturated when *Q* = 1 × 10^22^ cm^−3^ was applied. This corresponds to a total oxidation time of 410 s.

Each time after applying *Q* = −1 × 10^21^ cm^−3^/+1 × 10^21^ cm^−3^, the out-of-plane x-ray diffraction (XRD) pattern of the device was recorded as shown in Fig. 2 (B and E). Upon reduction (Fig. 2B), several diffraction peaks of reduced CeO_2−δ_, labeled b, c, and d appear sequentially in addition to the diffraction peak of CeO_2_ (labeled a). These phases are heteroepitaxially grown on the YSZ substrate. The x-ray reciprocal space mappings of the CeO_2_-based thermal switch reveal that all the CeO_2−δ_ (a, b, c, and d) crystals are isotropic (fig. S4, A to C). Thus, all the CeO_2−δ_ crystals are fully relaxed, and there is no epitaxial strain at the film-substrate heterointerface. This was confirmed by the high-angle annular dark-field (HAADF)–scanning transmission electron microscopy (STEM) observations (fig. S5, A and B). Misfit dislocations (yellow arrows) resulting from the lattice mismatch (~+5%) are visualized (text S2). We then analyzed the homologous phases of Ce*_n_*O_2*n*−2_ (*n* ≥ 4, integer) as a function of δ in CeO_2−δ_. Using the reported lattice parameters (*31*) of CeO_2_, CeO_1.832_, and CeO_1.735_, we found a linear relationship between the δ and the lattice parameter (text S3). The linear relationship was then extended to δ = 0.5. The linear relationship we clarified revealed that the a, b, c, and d phases are CeO_2_ (*n* = ∞), Ce_9_O_16_, Ce_3_O_5_, and Ce_2_O_3_, respectively. Upon oxidation (Fig. 2E), the diffraction peak of reduced CeO_2−δ_ (c phase) shifts to a larger *q*_z_/2π value and eventually disappears. We further clarified that the reduction of Ce^4+^ to Ce^3+^ occurs after the electrochemical reduction treatment (text S4).

If the electrochemical reduction of CeO_2_ obeys Faraday’s laws of electrolysis, then the following electrochemical reaction occurs

$${\text{CeO}}_{2}+\text{2}\delta e^{-}\to {\text{CeO}}_{2-\delta }+{\delta O}^{2-}\;\;\left(0\leq \delta \leq 0.5\right)$$

This indicates that the CeO_2_ becomes Ce_2_O_3_ when the total *Q* reaches −2.6 × 10^22^ cm^−3^. However, the XRD results show that there are several phases, CeO_2_ (a) and CeO_2−δ_ (b, c, and d), probably because the formation energy of homologous phases of Ce_n_O_2n−2_ (*n* ≥ 4, integer) is close to each other and spontaneous oxidation occurs at 280°C in the air.

### Thermal stability of the reduced cerium oxide film

**Fig. 3.**
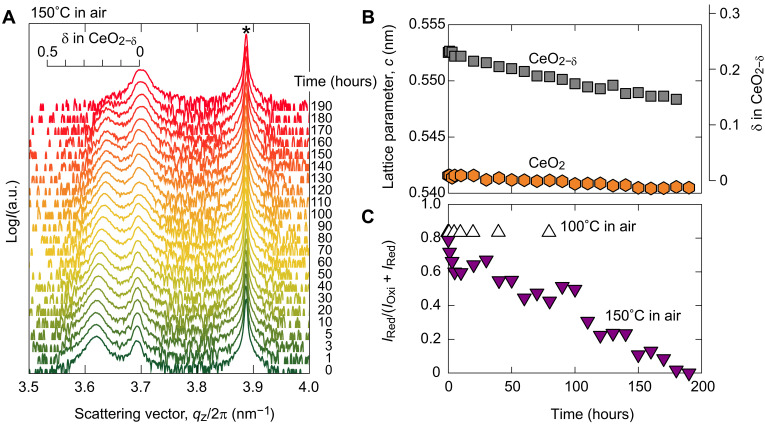
Thermal stability of the reduced CeO_2_-based thermal switch. (**A**) Change in the out-of-plane XRD patterns of the reduced CeO_2_-based thermal switch while kept at 150°C in the air. After the reduction (*Q* = −1.5 × 10^22^ cm^−3^, 0 hours), the diffraction peak of 002 CeO_2−δ_ (δ ~ 0.24, b) is seen together with 002 CeO_2_ (a). The peak position of b shifts to the higher *q*_z_/2π side, and the peak b becomes weaker with time. On the other hand, peak a becomes stronger with time. After 190 hours, the peak b disappears. (**B**) Change of lattice parameter, *c*, of the CeO_2_ (a) and CeO_2−δ_ (b) with time. The *c* of the b phase gradually decreases with time while that of the a phase is almost constant. (**C**) XRD peak intensity ratio [*I*_**b**_/(*I*_**a**_ + *I*_**b**_)], which reflects the volume fraction of the b phase. The results at 100°C in air are also shown. At 150°C, the intensity ratio is ~0.8 at the beginning and decreases almost linearly with time, reaching zero after 190 hours. In contrast, the intensity ratio is almost constant at 100°C. a.u., arbitrary units.

When we place the reduced sample on the 280°C heater without applying a negative current, the film oxidizes within 10 min. At lower temperatures (100°C), such spontaneous oxidation does not occur. Figure 3A summarizes the change in the out-of-plane XRD patterns of the reduced CeO_2_-based thermal switch while held at 150°C in the air. After the reduction (*Q* = −1.5 × 10^22^ cm^−3^, 0 hours), the diffraction peak of 002 CeO_2−δ_ (δ ~ 0.24, b) is seen together with 002 CeO_2_ (a). The peak position of b shifts to a higher *q*_z_/2π side, and the peak b becomes weaker with time. On the other hand, peak a becomes strong with time. After 190 hours, peak b disappears. The lattice parameter *c* of the b phase gradually decreases with time, while the a phase is almost constant (Fig. 3B). We calculated the XRD peak intensity ratio [*I*_b_/(*I*_a_ + *I*_b_)], which reflects the volume fraction of the b phase (Fig. 3C). At 150°C, the intensity ratio is ~0.8 at the beginning, whereas it decreases almost linearly with time, reaching zero after 190 hours. In contrast, the intensity ratio is almost constant at 100°C. Thus, spontaneous oxidation occurs at 280°C during electrochemical reduction, and this is one of the origins of the coexistence of a, b, c, and d phases during reduction.

### Thermal conductivity of oxidized/reduced cerium oxide films

**Fig. 4.**
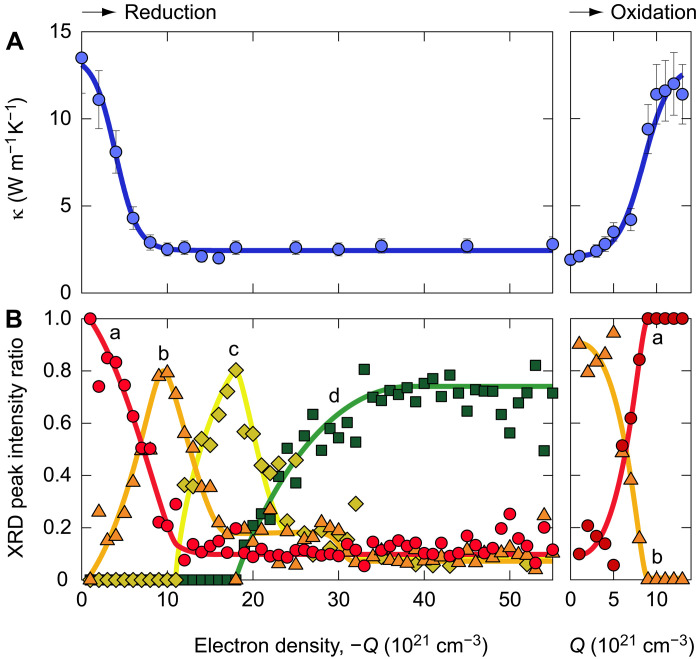
Thermal conductivity modulation of the CeO_2_-based thermal switches. (**A**) Thermal conductivity (κ) of the CeO_2_ layer as a function of electron density (*Q*). The fully oxidized CeO_2_ layer (a) shows a higher κ of ~13 W m^−1^ K^−1^. The κ decreases markedly with *Q* and reaches ~2.5 W m^−1^ K^−1^ at *−Q* ~ 10 × 10^21^ cm^−3^. Thereafter, κ is nearly constant (~2.5 W m^−1^ K^−1^). On the other hand, when oxidized, κ increases markedly and reaches ~12.5 W m^−1^ K^−1^. (**B**) XRD peak intensity ratio. During reduction, the peak intensity ratio of a phase decreases markedly with *Q* and reaches ~0.1 when *Q* ~ 10 × 10^21^ cm^−3^. On the other hand, the b phase increases and peaks around *−Q* ~ 10 × 10^21^ cm^−3^ and then decreases. Then, the c phase increases and has a peak around *−Q* ~ 18 × 10^21^ cm^−3^ and then decreases. Last, the d phase increases and saturates around *−Q* ~ 35 × 10^21^ cm^−3^. During oxidation, the intensity of the b phase decreases markedly, and the intensity of the a phase increases markedly. The κ is nearly correlated with the volume fraction of the a phase.

Next, we measured the κ of the CeO_2_-based thermal switch by a time-domain thermoreflectance (TDTR) method at room temperature. Details of the TDTR measurements are described in text S5 and Materials and Methods. The typical κ of the as-grown CeO_2_-based thermal switch was 13.5 W m^−1^ K^−1^ (fig. S8), which was similar to that of bulk CeO_2_ (*25*–*27*), reflecting the high crystal quality of the CeO_2_ film. The decay of the TDTR phase signal of the oxidized sample is faster than that of the reduced sample (fig. S9). We simulated the TDTR decay curves and determined the κ of the CeO_2_ films during reduction/oxidation (Fig. 4A). During the reduction, the κ decreases markedly with *Q* and reaches ~2.5 W m^−1^ K^−1^ when *Q* ~ −10 × 10^21^ cm^−3^. Thereafter, κ is nearly constant (~2.5 W m^−1^ K^−1^). This is larger than Khafizov’s calculation [1.2 W m^−1^ K^−1^ (*34*)]. On the other hand, when oxidized, the κ increases markedly and reaches ~12.5 W m^−1^ K^−1^ when *Q* ~ 11 × 10^21^ cm^−3^. To clarify the origin of the κ modulation, we calculated the XRD peak intensity ratio of a, b, c, and d phases, which reflects the volume fraction of each phase (Fig. 4B). During the reduction, the peak intensity ratio of a phase decreases drastically with *Q*, reaching ~0.1 when *Q* ~ −10 × 10^21^ cm^−3^. On the other hand, the b phase increases and peaks around *Q* ~ −10 × 10^21^ cm^−3^ and then decreases. Then, the c phase increases and has a peak around *Q* ~ −18 × 10^21^ cm^−3^ and then decreases. Last, the d phase increases and saturates around *Q* ~ −35 × 10^21^ cm^−3^. During oxidation, the intensity of the b phase decreases markedly, and the intensity of the a phase increases markedly. These results indicate that the κ correlates only with the volume fraction of the a phase.

### Cyclability of the thermal switches

**Fig. 5.**
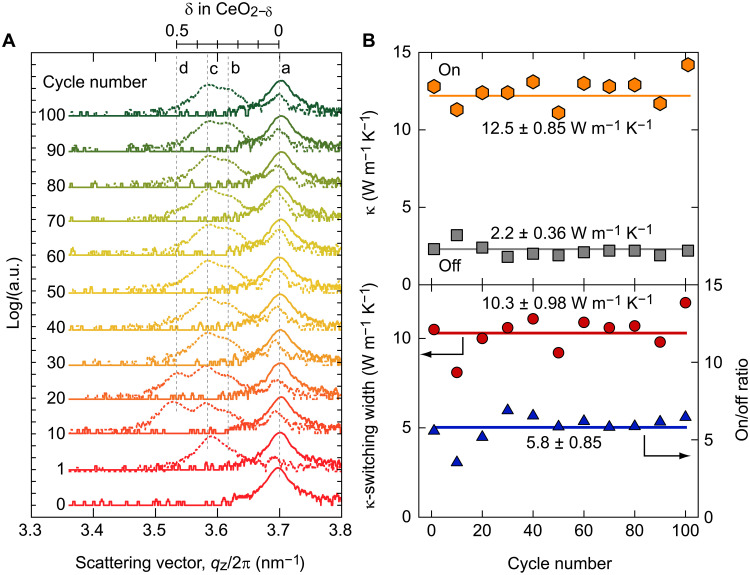
Cyclic properties of the CeO_2_-based thermal switch. (**A**) Out-of-plane XRD patterns of the on-state (solid lines) and off-state (dashed lines) after each cycling. In the on-state, only the diffraction peak of the a phase (CeO_2_) is observed, while in the off-state, diffraction peaks of reduced b to d phases with weak a phase are observed in all cases. (**B**) Changes in the thermal conductivity (κ) of the CeO_2_ layer as a function of the number of redox cycles. The average κ is 2.2 ± 0.36 W m^−1^ K^−1^ for the reduced off-state and 12.5 ± 0.85 W m^−1^ K^−1^ for the oxidized on-state. The average on/off κ*-*ratio of the CeO_2_ layer is 5.8 ± 0.85, and the κ-switching width is 10.3 ± 0.98 W m^−1^ K^−1^.

Next, the cyclability of the thermal switches was tested. The electrochemical reduction/oxidation treatments were performed by applying a constant current of −10 μA/+10 μA. These treatments were performed by applying electron densities *Q* of −1.5 × 10^22^ cm^−3^ for reduction and *Q* of 0.9 × 10^22^ cm^−3^ for oxidation. Treatments were repeated for 100 cycles. We measured changes in the out-of-plane XRD patterns of the CeO_2_ film during reduction (off-state) and oxidation (on-state) (Fig. 5A). In the on-state, only the a-phase (CeO_2_) diffraction peak is observed, whereas in the off-state, diffraction peaks of reduced b, c, and d phases with weak a phase are observed in all cases. It is remarkable that we did not observe severe degradation of the crystal structure until 100 times cycling. The TDTR decay curves of the CeO_2_-based thermal switches were measured every 10 cycles at room temperature (fig. S10), and the κ was calculated (Fig. 5B). The average κ was 2.2 ± 0.36 W m^−1^ K^−1^ for the reduced off-state and 12.5 ± 0.85 W m^−1^ K^−1^ for the oxidized on-state. The on/off κ-ratio of the CeO_2_ layer was 5.8 ± 0.85, and the κ*-*switching width was 10.3 ± 0.98 W m^−1^ K^−1^. The switching of crystallographic phases and κ remained stable for at least 100 cycles. This excellent cyclability of the thermal switch would be due to the crystallographic similarity of CeO_2_ and CeO_2−δ_ homologous phases.

### Thickness dependence of the switching properties

**Fig. 6.**
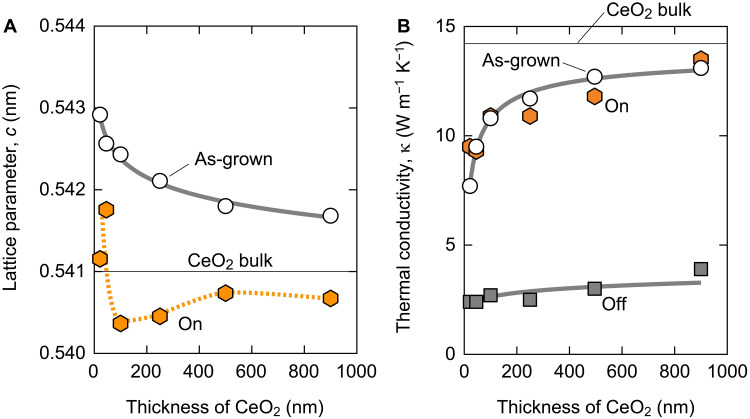
Effect of the thickness of CeO_2_ films. (**A**) Change of lattice parameter, *c*, of as-grown and oxidized CeO_2_ films with thickness. The lattice parameter decreases with thickness when the thickness is thin (~100 nm). (**B**) Variation of κ of the CeO_2_-based thermal switch measured at room temperature with CeO_2_ thickness. The κ increases markedly with thickness in both the as-grown and on-state when the thickness is thin.

To clarify the effect of CeO_2_ thickness on the thermal switch performance, we fabricated several thermal switches with different CeO_2_ film thicknesses. Electrochemical reduction of the thermal switches was performed by applying a constant dc current of −10 μA until the total *Q* reached −1.5 × 10^22^ cm^−3^. On the other hand, the oxidation treatment was performed by applying a constant dc current of +10 μA until the total *Q* reached 0.9 × 10^22^ cm^−3^. Figure S11 summarizes the out-of-plane XRD patterns of the resulting thermal switches. The diffraction peak of a phase (CeO_2_) of the as-grown samples shifts to a higher *q*_z_/2π side with increasing thickness. After the reduction treatment, a, b, and c phases are randomly distributed most likely due to the contribution of spontaneous oxidation. After the oxidation treatment, only a phase diffraction peaks were observed, and the peaks slightly shifted to a higher *q*_z_/2π side with increasing thickness. Using the XRD patterns, we extracted the lattice parameter of the as-grown and oxidized CeO_2_ films (Fig. 6A). In the case of the as-grown films, the lattice parameter was larger than that of the bulk (0.541 nm) and decreased with thickness when the thickness is thin (~100 nm). This could be due to the oxygen deficiency of the thinner films. Figure 6B summarizes the variation of the κ of the CeO_2_-based thermal switch measured at room temperature with the CeO_2_ thickness. The κ increases markedly with thickness in both the as-grown and on-state when the thickness is thin, most likely due to an increase in oxygen concentration. Thereafter, the κ gradually increases and approaches to the bulk value (14 W m^−1^ K^−1^). From these results, the use of thick film or less oxygen-deficient film is appropriate for realizing solid-state electrochemical thermal switches with a large κ-switching width.

## DISCUSSION

**Fig. 7.**
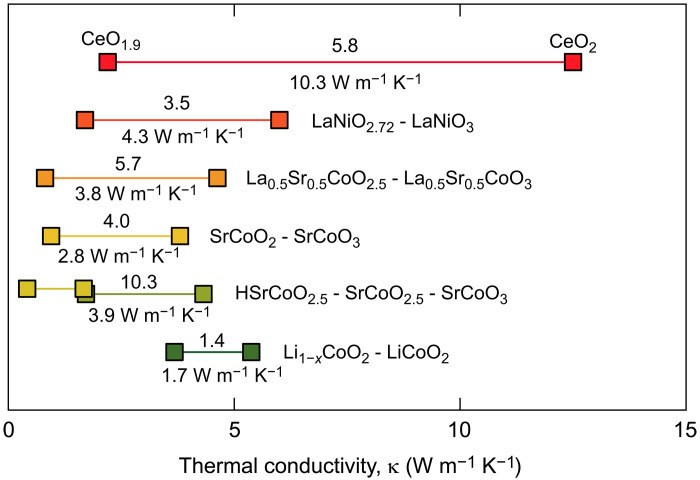
Comparison of the thermal conductivity switching for the reported oxide-based electrochemical thermal switches. LiCoO_2_/Li_1−*x*_CoO_2_ [Cho *et al*. (*10*)], SrCoO_3_/SrCoO_2.5_/HSrCoO_2.5_ [Lu *et al*. (*12*)], SrCoO_3_/SrCoO_2_ [Yang *et al*. (*14*–*16*)], La_0.5_Sr_0.5_CoO_3_/La_0.5_Sr_0.5_CoO_2.5_ [Zhang *et al*. (*17*)], LaNiO_3_/LaNiO_2.72_ [Bian *et al*. (*18*)], and CeO_2_/CeO_1.9_ (this study). The value above each line is the on/off κ-ratio, and the value below each line is the κ-switching width. The CeO_2_-based thermal switch shows a rather large on/off κ-ratio of 5.8 and a very large κ-switching width of ~10.3 W m^−1^ K^−1^.

We have realized high-performance solid-state thermal switches with a relatively high on/off κ*-*ratio of 5.8 and a large κ*-*switching width of 10.3 W m^−1^ K^−1^ using abundant cerium oxide as the active material. This is an excellent performance compared to other values reported for oxide-based thermal switches (Fig. 7). In the oxidized on-state, the κ was about 12.5 W m^−1^ K^−1^, and κ decreased markedly to 2.2 W m^−1^ K^−1^ by electrochemical reduction. The oxidized state contains only one CeO_2_ phase, while the reduced state contains several CeO_2−δ_ phases (Ce*_n_*O_2*n*−2_, *n* = 9, 6, and 4). The switching of crystallographic phases and thermal conductivity were stable for at least 100 cycles. The present CeO_2_-based solid-state electrochemical thermal switches would have the potential for use in thermal management devices such as thermal displays.

## MATERIALS AND METHODS

### Fabrication of the CeO_2_-based thermal switches

CeO_2_ thin films were heteroepitaxially grown on (001) YSZ single-crystalline substrates (10 mm by 10 mm, 0.5-mm thickness) by pulsed laser deposition (KrF excimer laser, ~1.5 J cm pulse, 10 Hz). The substrate temperature was maintained at 800°C during the film growth. The oxygen pressure was 3 × 10^−3^ Pa. The deposition rate was approximately 10 nm min^−1^. Details of the CeO_2_ film growth are described elsewhere (*36*, *37*). After the CeO_2_ film growth, Pt films were sputtered on the CeO_2_ film surface (80 nm) and the backside (30 nm) of the YSZ substrate. Last, the substrate was cut into four pieces (5 mm by 5 mm).

### Characterization of the crystal structure

The surface morphology of the as-grown CeO_2_ films was evaluated using reflection high-energy electron diffraction patterns and topographic atomic force microscopy observations. We used high-resolution XRD (Cu Kα_1_ radiation, λ = 0.154059 nm, ATX-G, Rigaku Co.) to measure x-ray reflectivity, out-of-plane Bragg diffraction patterns, out-of-plane x-ray rocking curves, and reciprocal space mapping of the CeO_2_-based thermal switches. To further clarify the crystal structure changes, the atomic arrangements were observed by HAADF STEM (TEM/STEM, JEM-ARM200F, JEOL Co.) operating at 200 kV.

### Redox treatment

A CeO_2_-based thermal switch (5 mm by 5 mm) was placed on a Pt-coated glass substrate and heated at 280°C in air. Then, we applied a constant current of −10 μA/+10 μA for reduction/oxidation. We applied the current for 40 s to apply *Q* = 1 × 10^21^ cm^−3^ and repeated the current application until total *Q* reached 5 × 10^22^ cm^−3^. The diffusion coefficient of O^2−^ ions in CeO_2_ crystal at 280°C is ~5.5 × 10^−13^ cm^2^ s^−1^ from the literature (*38*), and the required time for the spontaneous diffusion of O^2−^ ions for 100 nm is calculated to 180 s. After applying the current, the sample was immediately cooled to room temperature.

### Measurements of the thermal conductivity (κ)

The κ value of CeO_x_ films perpendicular to the substrate surface was measured by TDTR (PicoTR, NETZSCH Japan). The top Pt film was used as the transducer. The decay curves of the TDTR signals were simulated to obtain κ. The specific heat capacities of the layers used for the TDTR simulation were Pt: 133 J kg^−1^ K^−1^; CeO_2_: 358 J kg^−1^ K^−1^; and YSZ: 460 J kg^−1^ K^−1^ (table S1). Details of our TDTR method are described elsewhere (*14*). Regarding the treatment of the κ values, since there were several uncertainties such as the position of the baseline, position of the time zero, and the noise of the signal, error bars of ±15% of the obtained values were used.

## Supplementary Materials

### This PDF file includes:

Supplementary Text Figs. S1 to S11 Table S1 References
