# Supplementary material for: High-performance solid-state electrochemical thermal switches with earth-abundant cerium oxide
Source: Sci Adv. 2025 Jan 1;11(1):eads6137. doi: 10.1126/sciadv.ads6137 (PMC11691636; doi:10.1126/sciadv.ads6137)
Supplement: Supplementary file 1 — Supplementary Text Figs. S1 to S11 Table S1 References [file sciadv.ads6137_sm.pdf]

Science Advances
AAAS

Supplementary Materials for

High-performance solid-state electrochemical thermal switches with earth-abundant cerium oxide

Ahrong Jeong *et al.*

Corresponding author: Hiromichi Ohta, hiromichi.ohta@es.hokudai.ac.jp

Sci. Adv. **11**, eads6137 (2025)
DOI: 10.1126/sciadv.ads6137

This PDF file includes:

Supplementary Text
Figs. S1 to S11
Table S1
References

S1. Fabrication of CeO_2-based solid-state electrochemical thermal switches

First, we fabricated CeO_2 epitaxial films on (001) oriented YSZ single crystal substrates by pulsed laser deposition (PLD) at 800 °C under an oxygen atmosphere (3×10^{-3} Pa). Details of the CeO_2 film growth are described elsewhere. (*36*, *37*) During the CeO_2 film growth in the PLD chamber, we monitored the reflection high-energy electron diffraction (RHEED, acceleration voltage: 25 kV, azimuth: <100>) patterns as shown in **Fig. S1A**. The rod-like RHEED pattern indicates that rather flat CeO_2 film is heteroepitaxially grown on the (001) YSZ substrate. The atomic force microscopy (AFM) topographic image of the film surface (**Fig. S1B**) confirms this conclusion. The film is composed of grains in the range of 30 – 100 nm. There are also many dark spots indicating threading dislocations. (*39*) **Figure S1C** shows the X-ray reflectivity of the CeO_2 film on (001) YSZ single crystal substrate. The calculated result shows that the root mean square roughness (R_{rms}) of the CeO_2 film is 0.72 nm and the thickness is 103 nm calculated from the Kiessig fringes.

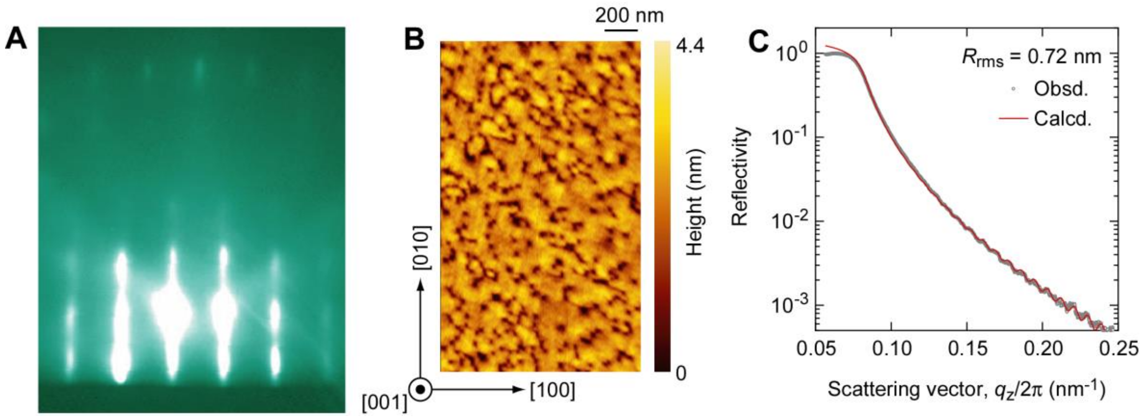

Figure S1. Surface morphology of the CeO_2 film on (001) YSZ substrate. (**A**) RHEED pattern (azimuth: <100>), (**B**) Topographic AFM image, (**C**) XRR pattern.

After checking the XRR, we deposited Pt films on the surface of the CeO_2 film and the back side of the YSZ substrate by DC magnetron sputtering, and then measured the out-of-plane XRD patterns of the film. **Figure S2A** shows the out-of-plane XRD pattern of the CeO_2-based thermal switch. Intense diffraction peaks of 002 and 004 CeO_2 are seen with 002 YSZ. As shown in the magnified pattern (**Fig. S2B**), Pendellösung fringes are observed, indicating a strong 00*l* orientation of the CeO_2 film. The film thickness that extracted from the Pendellösung fringes is 103 nm, which agrees well with the thickness extracted from the Kiessig fringes (**Fig. S1C**). The full width at half maximum (FWHM) of the X-ray rocking curve of 002 CeO_2 is 0.026 degrees, nearly equal to the resolution (**Fig. S2C**), confirming the strong 00*l* orientation of the film. We also measured the X-ray reciprocal space mapping (RSM) around 113 diffraction spots of CeO_2 and YSZ (**Fig. S2D**). The RSM indicates that a fully relaxed CeO_2 was grown on the (001) YSZ substrate.(*40*) The lateral grain size (*D*) was ~60 nm, which was estimated from the diffraction spot of 113 CeO_2 (**Fig. S2E**), in good agreement with the AFM observation (**Fig. S1B**).

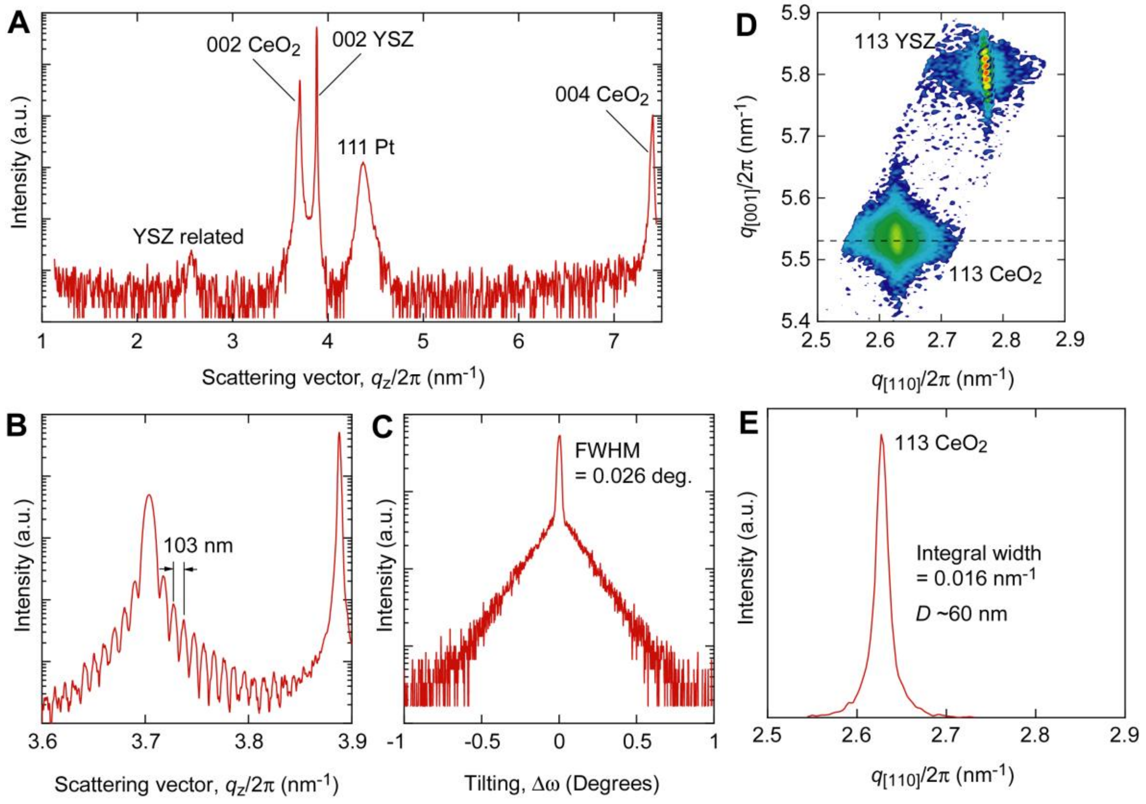

Figure S2. X-ray diffraction analyses of the CeO_2-based thermal switch. (**A**) Out-of-plane X-ray Bragg diffraction pattern, (**B**) Magnified XRD pattern of (**A**), (**C**) Out-of-plane X-ray rocking curve, (**D**) RSM, (**E**) Cross section pattern of (**D**) dotted line.

The layered structure of Pt/CeO_2/YSZ (**Fig. S3**) is visualized by the elemental mapping of the HAADF-STEM images (different sample).

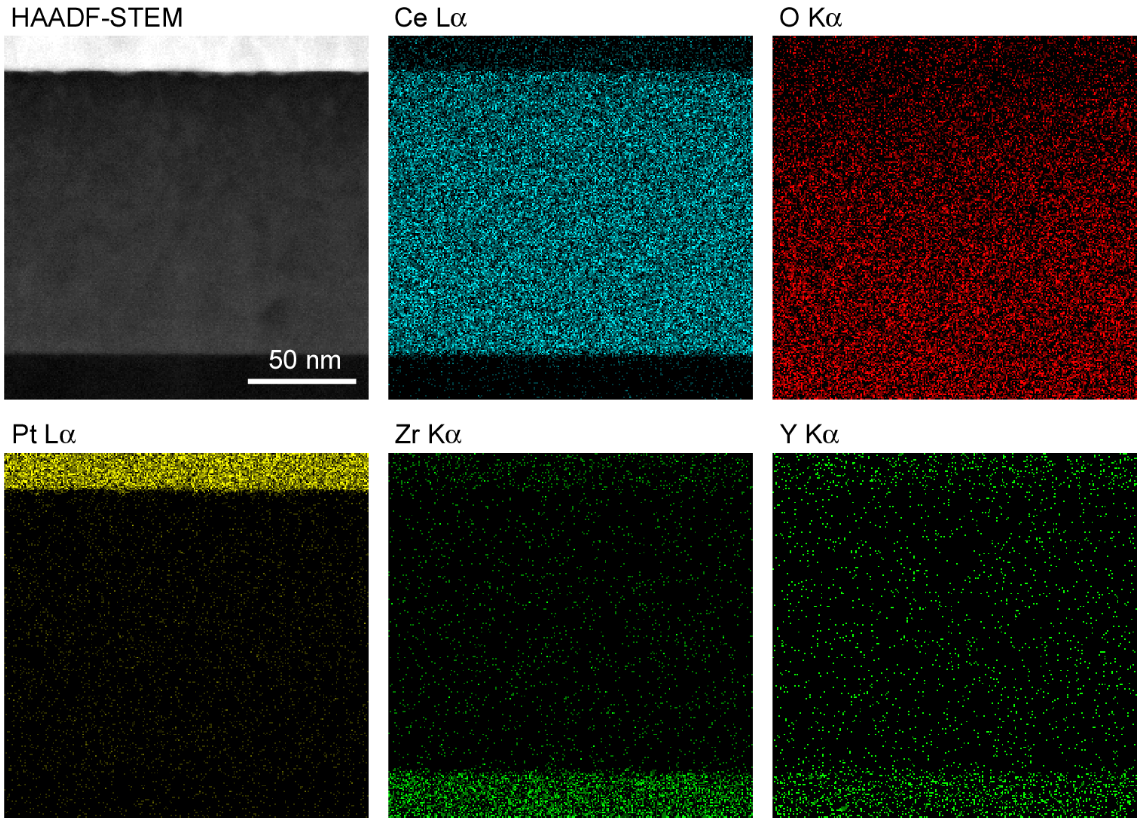

Figure S3. EDS elemental mapping of the CeO_2-based thermal switch. The layered structure composed of Pt/CeO_2/YSZ is visible.

S2. Electrochemical reduction/oxidation of the CeO_2-based thermal switches

The electrochemical reduction/oxidation treatments were performed by applying a constant current of −10 μA/+10 μA (**Figs. 2A** and **2D**). These treatments were performed at an electron density Q of 1×10^{21} cm^{-3} in each step. During electrochemical reduction (**Fig. 2A**), the absolute value of the applied voltage remains almost constant (~3.3 V). We repeated the reduction treatment until the total Q reached -5.5×10^{22} cm^{-3}, corresponding to a total reduction time of 2255 s. In contrast, the voltage required for initial oxidation (**Fig. 2D**) was negative, reflecting spontaneous oxidation. When Q exceeds ~7×10^{21} cm^{-3}, the required voltage increases dramatically from ~0 V to ~5 V. The required voltage became saturated when $Q = 1 \times 10^{22}$ cm^{-3} was applied. After that, the required voltage decreased with time and became constant around 3.3 V.

In addition to the out-of-plane XRD measurements (**Figs. 2B** and **2E**), we measured X-ray reciprocal space mapping (RSM) of the CeO_2-based thermal switches after oxidation (**Fig. S4A**), after weak reduction (**Fig. S4B**), and after complete reduction (**Fig. S4C**). The RSM results indicate that all the Ce_nO_{2n-2} ($n \geq 4$, integer, marked as **a**, **b**, **c**, and **d**) crystals are isotropic. Thus, all the Ce_nO_{2n-2} crystals are fully relaxed and there is no epitaxial strain at the film-substrate heterointerface. This was confirmed by the HAADF-STEM observations (**Figs. S5A** and **S5B**). Misfit dislocations (yellow arrows) resulting from the lattice mismatch (~+5%) are visualized.

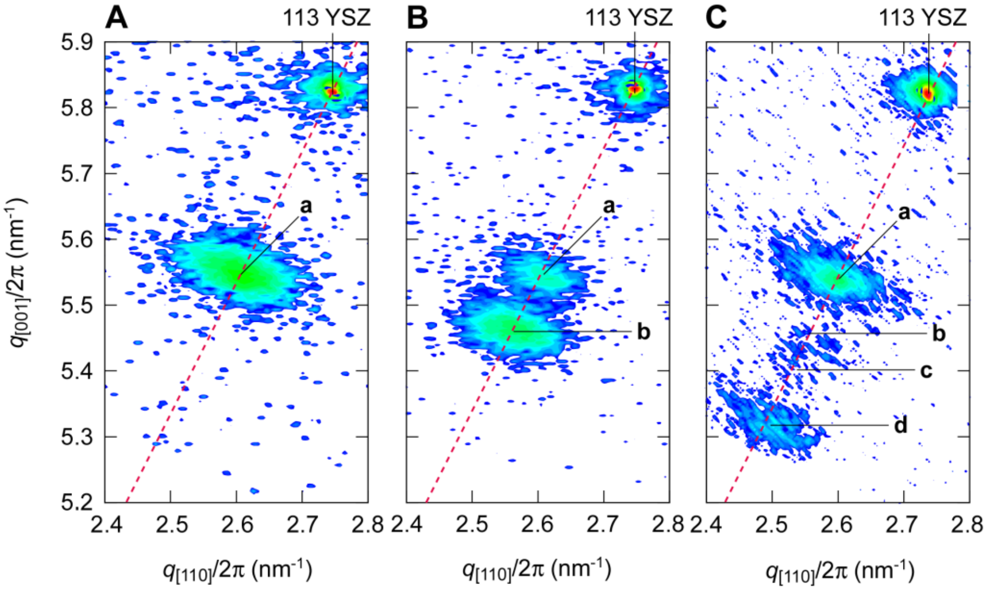

Figure S4. X-ray reciprocal space mappings of the CeO_2-based thermal switch. (**A**) After oxidation ($Q = 1.0 \times 10^{22}$ cm^{-3}), (**B**) after weak reduction ($Q = -1.4 \times 10^{22}$ cm^{-3}), (**C**) after complete reduction ($Q = -5.5 \times 10^{22}$ cm^{-3}). Red dotted lines with a slope of 2 are drawn across the diffraction peak of 113 YSZ; The crystal is isotropic when the diffraction peak is on the red dotted line. Diffraction peaks of **a**, **b**, **c**, and **d** phases are on the lines.

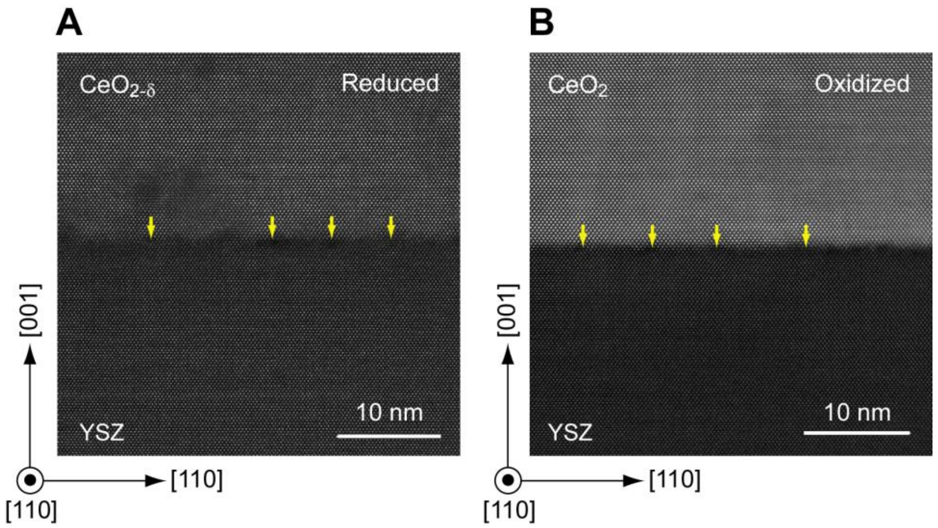

Figure S5. HADF-STEM images. (**A**) after reduction (**b** phase), (**B**) after oxidation (**a** phase). Misfit dislocations (yellow arrows) resulting from the lattice mismatch (~+5%) are visualized.

S3. Homologous phases of Ce_nO_{2n-2} ($n \geq 4$, integer)

To analyze the homologous phases of **a**, **b**, **c**, and **d**, we plotted the lattice parameter (fluorite structure) of Ce_nO_{2n-2} ($n \geq 4$, integer) as a function of δ in $CeO_{2-\delta}$. Using the reported lattice parameters (*31*) of CeO_2, $CeO_{1.832}$, and $CeO_{1.735}$, we found a linear relationship between the δ and the lattice parameter (**Fig. S6A**). The linear relationship was then extended to $\delta = 0.5$. Using the linear relationship, we clarified that the **a**, **b**, **c**, and **d** phases are CeO_2 ($n = \infty$), Ce_9O_{16}, Ce_3O_5, and Ce_2O_3, respectively. We also draw the crystal structures of CeO_2, $Ce_{11}O_{20}$, Ce_7O_{12}, and Ce_2O_3 using the crystallographic data reported by Kummerle *et al*.(*31*) The VESTA program (*41*) was used for the drawing and simulating the powder diffraction patterns (**Fig. S6B**). Except for CeO_2, the displacement of Ce and O atoms is large. This would reflect the low thermal conductivity of Ce_nO_{2n-2} ($n \geq 4$, integer) except CeO_2.

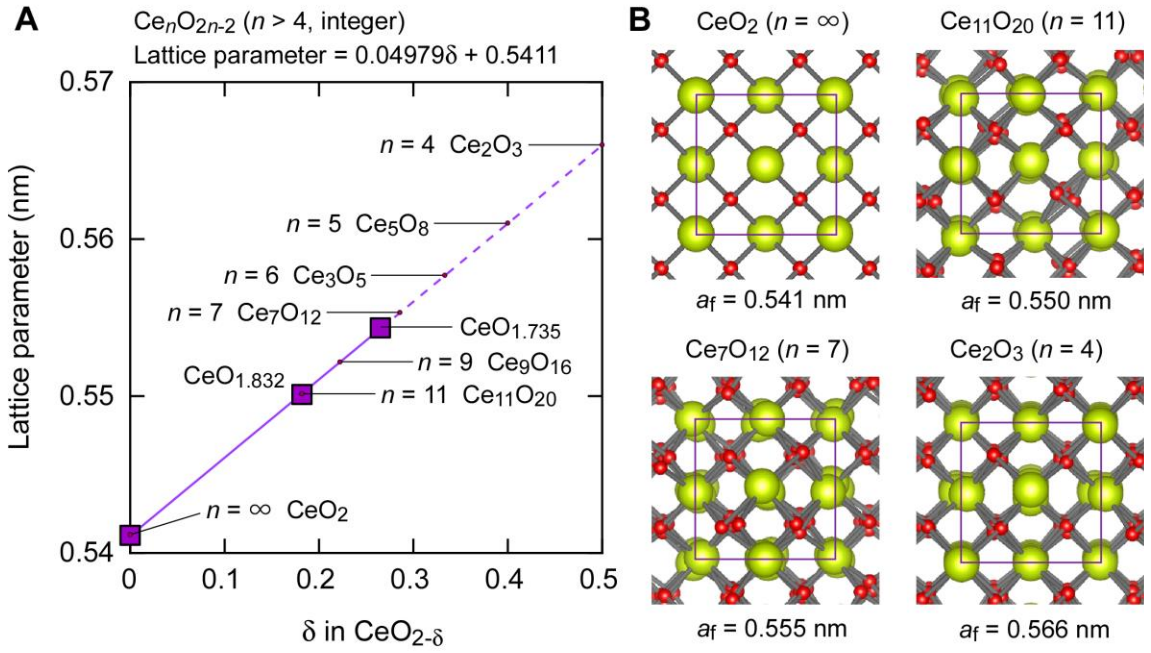

Figure S6. Homologous phases of Ce_nO_{2n-2}. (**A**) Lattice parameters (fluorite structure) of Ce_nO_{2n-2} ($n \geq 4$, integer) as a function of δ in $CeO_{2-\delta}$. First, the lattice parameters of the reported CeO_2, $CeO_{1.832}$, and $CeO_{1.735}$ were plotted. Then, the solid line was drawn with the least square fit. The line then was extended to $\delta = 0.5$ by assuming a linear relationship. Multiple *n*-values are indicated on the line. (**B**) Crystal structures of CeO_2, $Ce_{11}O_{20}$, Ce_7O_{12}, and Ce_2O_3, that drawn using the crystallographic data reported by Kummerle *et al*.(*31*) The VESTA program (*41*) was used to draw and simulate the powder diffraction patterns. (Yellow balls: Ce, Red balls: Oxygen)

S4. STEM-EELS analysis of the CeO_2-based thermal switches

To clarify that the reduction of Ce^{4+} to Ce^{3+} occurs after the electrochemical reduction treatment, we performed the STEM-EELS analyses (**Fig. S7**) of the weakly reduced sample ($Q = -1.0 \times 10^{22}$ cm^{-3}). It is known that the intensity ratio of M_5/M_4 of Ce $M_{4,5}$ edge EELS spectra reflects the Ce^{3+}/Ce^{4+} concentration in $CeO_{2-\delta}$ films (Ce^{4+}: M_5/M_4 = 0.90, Ce^{3+}: M_5/M_4 = 1.25)(*36*). Ideally, the equation of M_5/M_4 ratio = $0.9 + 0.7\delta$ ($0 \leq \delta \leq 0.5$) is established. The M_5/M_4 ratio was calculated from the positive part of the second derivative of the experimental spectra. The M_5/M_4 ratio of the reduced $CeO_{2-\delta}$ film was 0.99, while that of the oxidized CeO_2 was 0.90. Thus, δ in the CeO_2 film was estimated to be 0.13 (**b** phase). Note that the observed M_5/M_4 ratio was varied as the TEM sample thickness, since the reduced film was composed of **a** and **b** phases.

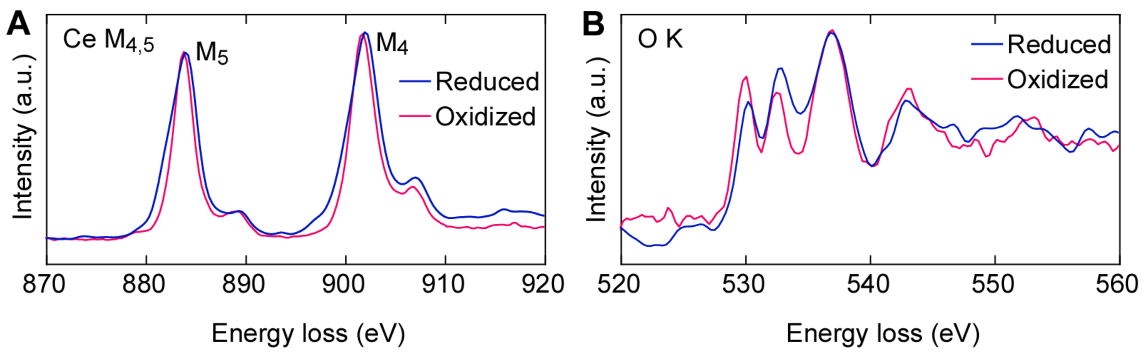

Figure S7. EELS of the CeO_2-based thermal transistor. (**A**) EELS spectra of Ce $M_{4,5}$ edges. (**B**) EELS spectra of O K edges. The M_5/M_4 ratio of the reduced $CeO_{2-\delta}$ film was 0.99, whereas that of the oxidized CeO_2 was 0.90.

S5. TDTR simulation of the CeO_2-based thermal switches

The cross-plane thermal conductivity of the CeO_2-based thermal switches was measured by the TDTR method at room temperature. We used commercially available TDTR equipment (PicoTR, PicoTherm Co.(*42*)). Sputtered Pt films were used as transducers. The samples were irradiated with femtosecond laser pulses (wavelength: 1550 nm; pulse duration: 0.5 ps; laser spot size: 45 μm), and the change in the reflectivity in the time domain was recorded with a probe laser (wavelength: 775 nm; pulse duration: 0.5 ps; laser spot size: 25 μm). The obtained thermoreflectance phase signals (**Figs. S8** – **S10**) were analyzed with a software package provided by the manufacturer (PicoTherm Co.(*42*)) using the physical properties listed in **Table S1**. We assumed that the specific heat capacity (C_p) of CeO_2 is always 358 J kg^{-1} K^{-1} and it is independent on the oxygen deficiency. To avoid the overestimation of κ, the Kapitza resistance (R_K) values at the Pt/CeO_2 interface lower than 4.0×10^{-9} m^2 K W^{-1} were used.

Table S1. Simulation condition of the TDTR decay curves for the CeO_2-based thermal switches. C_p: specific heat capacity, κ: thermal conductivity, R_K: interfacial thermal resistance.

	Pt	CeO_2	YSZ
Density (kg/m^3)	21500	7215	5770
C_p (J/kgK)	133	358	460
κ (W/mK)	46	----	2.00
R_K (m^2K/W)	$1.0 \times 10^{-9} - 4.0 \times 10^{-9}$		1.0×10^{-9}

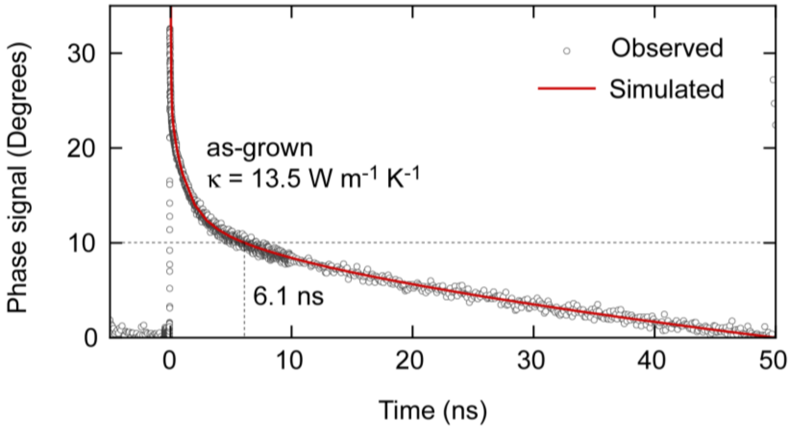

Figure S8. Typical TDTR decay curve of the as-grown CeO_2-based thermal transistor. The R_K at the Pt/CeO_2 interface was 4.0×10^{-9} m^2 K W^{-1}. The simulated curve reproduces the observed decay curve. The extracted κ of the CeO_2 layer is 13.5 W m^{-1} K^{-1}. The delay time at 10° is ~6.1 ns.

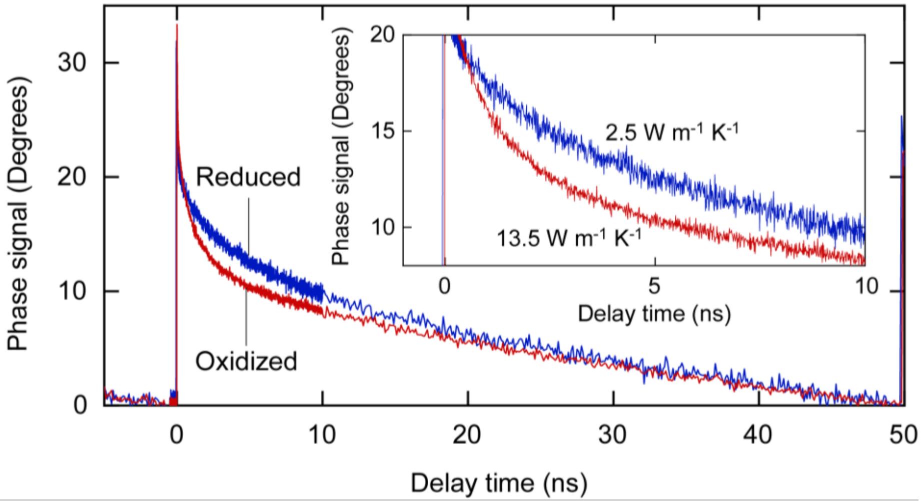

Figure S9. Thermal conductivity modulation of the CeO_2-based thermal switch after oxidation and reduction. TDTR decay curves of the CeO_2-based thermal switch measured at room temperature. The inset shows the magnified graph. The decay of the TDTR phase signal of the oxidized sample is faster than that of the reduced one.

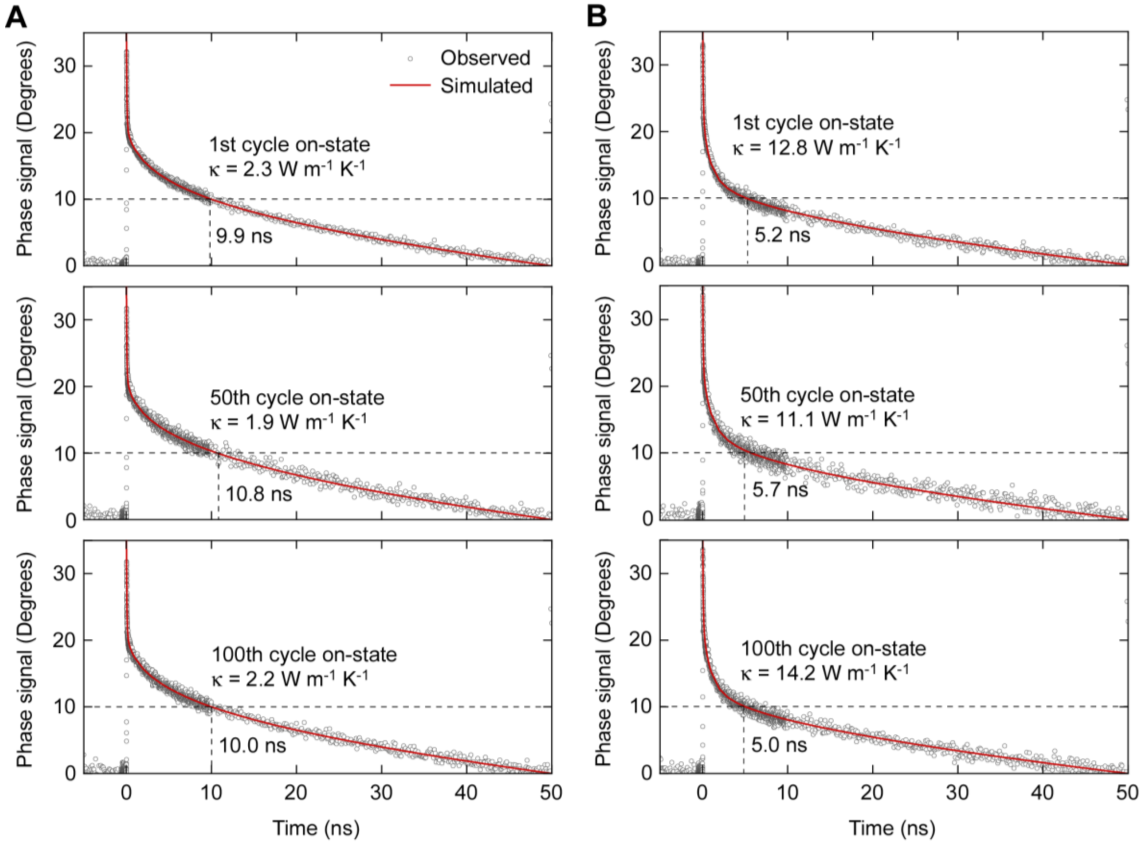

Figure S10. TDTR decay curves of the CeO_2-based thermal switch after 1st, 50th, and 100th cycles. (**A**) Reduced off-state, (**B**) Oxidized on-state. The delay time is shown for comparison.

S6. Thickness dependence of the CeO_2-based thermal switches

To clarify the effect of CeO_2 thickness on the thermal switch performance, we fabricated several thermal switches with different CeO_2 film thicknesses. Electrochemical reduction of the thermal switches was performed by applying a constant DC current of −10 μA until the total Q reached -1.5×10^{22} cm^{-3}. On the other hand, the oxidation treatment was performed by applying a constant DC current of +10 μA until the total Q reached 0.9×10^{22} cm^{-3}. **Figure S11** summarizes the out-of-plane XRD patterns of the resulting thermal switches. The diffraction peak of the **a** phase (CeO_2) of the as-grown samples shifts to a higher $q_z/2\pi$ side with increasing thickness. After the reduction treatment, **a**, **b**, and **c** phases are randomly distributed most likely due to the contribution of spontaneous oxidation. After the oxidation treatment, only **a** phase diffraction peaks are observed and the peaks shift slightly to a higher $q_z/2\pi$ side with increasing thickness.

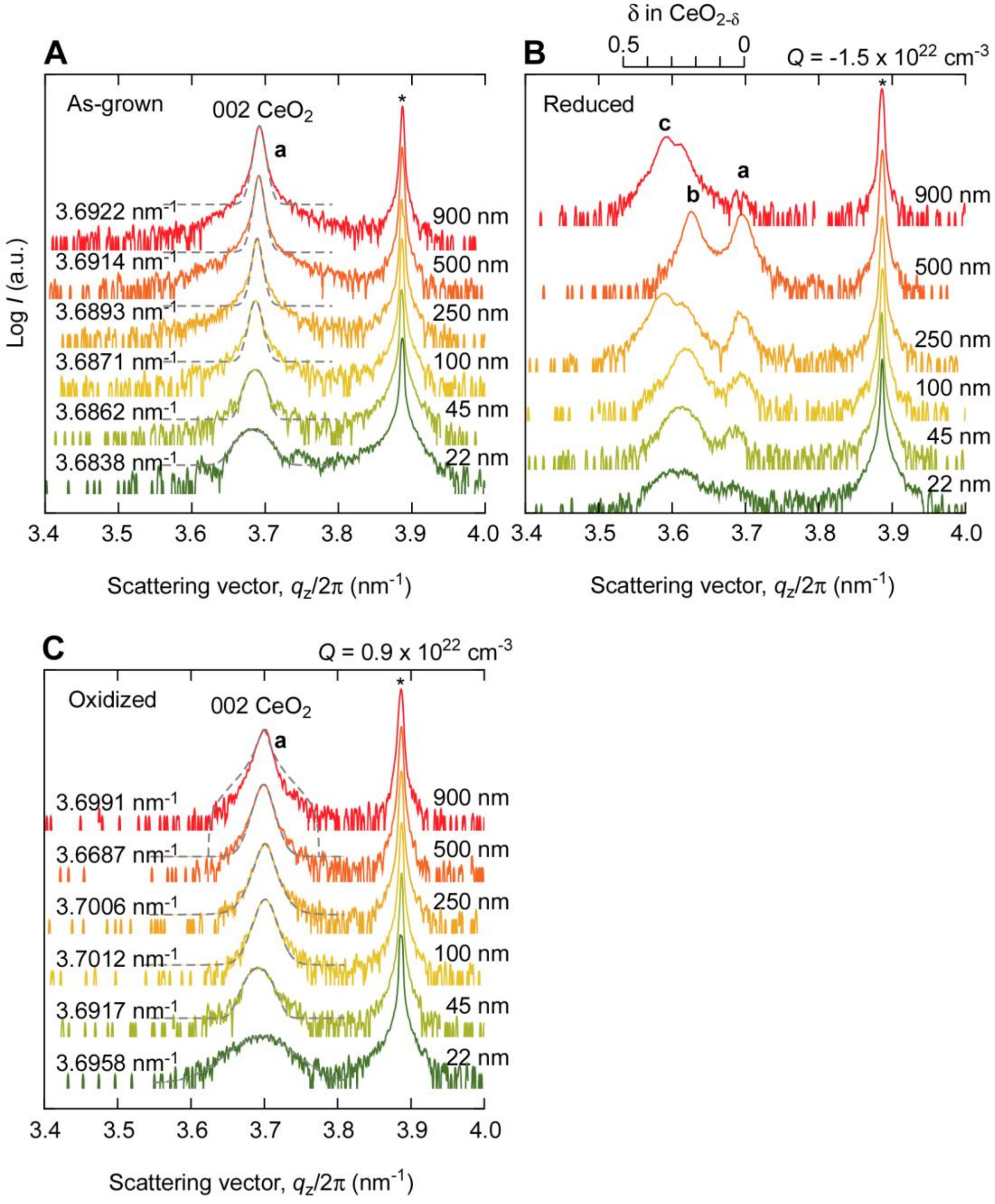

Figure S11. Out-of-plane XRD patterns of CeO_2-based thermal switches with varied CeO_2 thickness. (**A**) As-grown, (**B**) reduced ($Q = -1.5 \times 10^{22}$ cm^{-3}), (**C**) oxidized ($Q = 0.9 \times 10^{22}$ cm^{-3}). (**A**, **C**) The diffraction peak of **a**-phase (CeO_2) shifts to a higher $q_z/2\pi$ side with increasing thickness. (**B**) Although Q for the reduction is the same as -1.5×10^{22} cm^{-3}, **a**, **b**, and **c** phases are randomly distributed.

REFERENCES AND NOTES

1. Z. L. Bu, X. Y. Zhang, Y. X. Hu, Z. W. Chen, S. Q. Lin, W. Li, C. Xiao, Y. Pei, A record thermoelectric efficiency in tellurium-free modules for low-grade waste heat recovery. *Nat. Commun.* **13**, 237 (2022).

2. C. B. Vining, An inconvenient truth about thermoelectrics. *Nat. Mater.* **8**, 83–85 (2009).

3. J. He, T. M. Tritt, Advances in thermoelectric materials research: Looking back and moving forward. *Science* **357**, eaak9997 (2017).

4. T. Swoboda, K. Klinar, A. S. Yalamarthy, A. Kitanovski, M. Muñoz Rojo, Solid-State thermal control devices. *Adv. Electron. Mater.* **7**, 2000625 (2020).

5. B. Li, L. Wang, G. Casati, Negative differential thermal resistance and thermal transistor. *Appl. Phys. Lett.* **88**, 143501 (2006).

6. P. Ben-Abdallah, S. A. Biehs, Near-field thermal transistor. *Phys. Rev. Lett.* **112**, 044301 (2014).

7. G. Wehmeyer, T. Yabuki, C. Monachon, J. Wu, C. Dames, Thermal diodes, regulators, and switches: Physical mechanisms and potential applications. *Appl. Phys. Rev.* **4**, 041304 (2017).

8. C. N. Berglund, H. J. Guggenheim, Electronic properties of VO_2 near the semiconductor-metal transition. *Phys. Rev.* **185**, 1022–1033 (1969).

9. S. Lee, K. Hippalgaonkar, F. Yang, J. Hong, C. Ko, J. Suh, K. Liu, K. Wang, J. J. Urban, X. Zhang, C. Dames, S. A. Hartnoll, O. Delaire, J. Wu, Anomalously low electronic thermal conductivity in metallic vanadium dioxide. *Science* **355**, 371–374 (2017).

10. J. Cho, M. D. Losego, H. G. Zhang, H. Kim, J. Zuo, I. Petrov, D. G. Cahill, P. V. Braun, Electrochemically tunable thermal conductivity of lithium cobalt oxide. *Nat. Commun.* **5**, 4035 (2014).

11. A. Sood, F. Xiong, S. Chen, H. Wang, D. Selli, J. Zhang, C. J. McClellan, J. Sun, D. Donadio, Y. Cui, E. Pop, K. E. Goodson, An electrochemical thermal transistor. *Nat. Commun.* **9**, 4510 (2018).

12. Q. Lu, S. Huberman, H. Zhang, Q. Song, J. Wang, G. Vardar, A. Hunt, I. Waluyo, G. Chen, B. Yildiz, Bi-directional tuning of thermal transport in $SrCoO_x$ with electrochemically induced phase transitions. *Nat. Mater.* **19**, 655–662 (2020).

13. J. Zhou, Y. Wu, H. Kwon, Y. Li, X. Xiao, Y. Ye, Y. Ma, K. E. Goodson, H. Y. Hwang, Y. Cui, Heat conductor-insulator transition in electrochemically controlled hybrid superlattices. *Nano Lett.* **22**, 5443–5450 (2022).

14. Q. Yang, H. J. Cho, Z. Bian, M. Yoshimura, J. Lee, H. Jeen, J. Lin, J. Wei, B. Feng, Y. Ikuhara, H. Ohta, Solid-state electrochemical thermal transistors. *Adv. Funct. Mater.* **33**, 2214939 (2023).

15. Z. Bian, Q. Yang, M. Yoshimura, H. J. Cho, J. Lee, H. Jeen, T. Endo, Y. Matsuo, H. Ohta, Solid-state electrochemical thermal transistors with strontium cobaltite-strontium ferrite solid solutions as the active layers. *ACS Appl. Mater. Interfaces* **15**, 23512–23517 (2023).

16. M. Yoshimura, Q. Yang, Z. Bian, H. Ohta, Significant reduction in the switching time of solid-state electrochemical thermal transistors. *ACS Appl. Electron. Mater.* **5**, 4233–4239 (2023).

17. Y. Zhang, W. M. Postiglione, R. Xie, C. Zhang, H. Zhou, V. Chaturvedi, K. Heltemes, H. Zhou, T. Feng, C. Leighton, X. Wang, Wide-range continuous tuning of the thermal conductivity of $La_{0.5}Sr_{0.5}CoO_{3-\delta}$ films via room-temperature ion-gel gating. *Nat. Commun.* **14**, 2626 (2023).

18. Z. Bian, M. Yoshimura, A. Jeong, H. Li, T. Endo, Y. Matsuo, Y. Magari, H. Tanaka, H. Ohta, Solid-state electrochemical thermal switches with large thermal conductivity switching widths. *Adv. Sci.* **11**, e2401331 (2024).

19. J. A. Tomko, A. Pena-Francesch, H. Jung, M. Tyagi, B. D. Allen, M. C. Demirel, P. E. Hopkins, Tunable thermal transport and reversible thermal conductivity switching in topologically networked bio-inspired materials. *Nat. Nanotechnol.* **13**, 959–964 (2018).

20. L. Castelli, Q. Zhu, T. J. Shimokusu, G. Wehmeyer, A three-terminal magnetic thermal transistor. *Nat. Commun.* **14**, 393 (2023).

21. C. Liu, Y. Si, H. Zhang, C. Wu, S. Deng, Y. Dong, Y. Li, M. Zhuo, N. Fan, B. Xu, P. Lu, L. Zhang, X. Lin, X. Liu, J. Yang, Z. Luo, S. Das, L. Bellaiche, Y. Chen, Z. Chen, Low voltage-driven high-performance thermal switching in antiferroelectric $PbZrO_3$ thin films. *Science* **382**, 1265–1269 (2023).

22. M. Li, H. Wu, E. M. Avery, Z. Qin, D. P. Goronzy, H. D. Nguyen, T. Liu, P. S. Weiss, Y. Hu, Electrically gated molecular thermal switch. *Science* **382**, 585–589 (2023).

23. C. M. Hartquist, B. X. Li, J. H. Zhang, Z. H. Yu, G. X. Lv, J. Shin, S. V. Boriskina, G. Chen, X. H. Zhao, S. T. Lin, Reversible two-way tuning of thermal conductivity in an end-linked star-shaped thermoset. *Nat. Commun.* **15**, 5590 (2024).

24. J.-S. Zhou, J. B. Goodenough, B. Dabrowski, Transition from Curie-Weiss to enhanced Pauli paramagnetism in $R\mathrm{NiO}_3$ (*R* = La, Pr, ... Gd). *Phys. Rev. B* **67**, 020404(R) (2003).

25. K. Suzuki, M. Kato, T. Sunaoshi, H. Uno, U. Carvajal-Nunez, A. T. Nelson, K. J. McClellan, Thermal and mechanical properties of CeO_2. *J. Am. Ceram. Soc.* **102**, 1994–2008 (2019).

26. L. Malakkal, A. Prasad, D. Oladimeji, E. Jossou, J. Ranasinghe, B. Szpunar, L. Bichler, J. Szpunar, Atomistic and experimental study on thermal conductivity of bulk and porous cerium dioxide. *Sci. Rep.* **9**, 6326 (2019).

27. P. V. Mao, T. Arima, Y. Inagaki, K. Idemitsu, Evaluation of structural and thermal properties of $Ce_{1-y}Gd_yO_{2-x}$ solid solution. *Int. J. Thermophysics* **41**, 111 (2020).

28. S. P. Ray, D. E. Cox, Neutron-diffraction determination of crystal-structure of Ce_7O_{12}. *J. Solid State Chem.* **15**, 333–343 (1975).

29. M. Ricken, J. Nolting, I. Riess, Specific-heat and phase-diagram of nonstoichiometric ceria (CeO_{2-x}). *J. Solid State Chem.* **54**, 89–99 (1984).

30. I. Riess, R. Koerner, M. Ricken, J. Noelting, Nonstoichiometric phases in cerium oxide. *Solid State Ion.* **28-30**, 539–541 (1988).

31. E. A. Kümmerle, G. Heger, The structures of C-$Ce_2O_{3+\delta}$, Ce_7O_{12}, and $Ce_{11}O_{20}$. *J. Solid State Chem.* **147**, 485–500 (1999).

32. M. Zinkevich, D. Djurovic, F. Aldinger, Thermodynamic modelling of the cerium-oxygen system. *Solid State Ion.* **177**, 989–1001 (2006).

33. P. Gao, Z. Kang, W. Fu, W. Wang, X. Bai, E. Wang, Electrically driven redox process in cerium oxides. *J. Am. Chem. Soc.* **132**, 4197–4201 (2010).

34. M. Khafizov, I.-W. Park, A. Chernatynskiy, L. F. He, J. L. Lin, J. J. Moore, D. Swank, T. Lillo, S. R. Phillpot, A. El-Azab, D. H. Hurley, Thermal conductivity in nanocrystalline ceria thin films. *J. Am. Ceram. Soc.* **97**, 562–569 (2014).

35. D. G. Cahill, S. K. Watson, R. O. Pohl, Lower limit to the thermal-conductivity of disordered crystals. *Phys. Rev. B* **46**, 6131–6140 (1992).

36. H. Hojo, T. Mizoguchi, H. Ohta, S. D. Findlay, N. Shibata, T. Yamamoto, Y. Ikuhara, Atomic structure of a CeO_2 grain boundary: The role of oxygen vacancies. *Nano Lett.* **10**, 4668–4672 (2010).

37. J. Tam, B. Feng, Y. Ikuhara, H. Ohta, U. Erb, Crystallographic orientation-surface energy-wetting property relationships of rare earth oxides. *J. Mater. Chem. A* **6**, 18384–18388 (2018).

38. J. M. Floyd, Interpretation of transport phenomena in nonstoichiometric ceria. *Indian J. Technol.* **11**, 589–594 (1973).

39. H. Hojo, E. Tochigi, T. Mizoguchi, H. Ohta, N. Shibata, B. Feng, Y. Ikuhara, Atomic structure and strain field of threading dislocations in CeO_2 thin films on yttria-stabilized ZrO_2. *Appl. Phys. Lett.* **98**, 153104 (2011).

40. C. B. Gopal, M. García-Melchor, S. C. Lee, Y. Z. Shi, A. Shavorskiy, M. Monti, Z. X. Guan, R. Sinclair, H. Bluhm, A. Vojvodic, W. C. Chueh, Equilibrium oxygen storage capacity of ultrathin $CeO_{2-\delta}$ depends non-monotonically on large biaxial strain. *Nat. Commun.* **8**, 15360 (2017).

41. K. Momma, F. Izumi, *VESTA 3* for three-dimensional visualization of crystal, volumetric and morphology data. *J. Appl. Cryst.* **44**, 1272–1276 (2011).

42. S. H. Firoz, T. Yagi, N. Taketoshi, K. Ishikawa, T. Baba, Direct observation of thermal energy transfer across the thin metal film on silicon substrates by a rear heating-front detection thermoreflectance technique. *Meas. Sci. Technol.* **22**, 024012 (2011).
